# High Resolution Multiplex Confocal Imaging of the Neurovascular Unit in Health and Experimental Ischemic Stroke

**DOI:** 10.3390/cells12040645

**Published:** 2023-02-17

**Authors:** Jeffrey J. Lochhead, Erica I. Williams, Elizabeth S. Reddell, Emma Dorn, Patrick T. Ronaldson, Thomas P. Davis

**Affiliations:** 1 Department of Pharmacology, College of Medicine, University of Arizona, Tucson, AZ 85724, USA; 2 Department of Pharmacology and Toxicology, College of Pharmacy, University of Arizona, Tucson, AZ 85721, USA

**Keywords:** neurovascular unit, endothelial cells, astrocytes, pericytes, neurons, microglia, ischemic stroke

## Abstract

The neurovascular unit (NVU) is an anatomical group of cells that establishes the blood–brain barrier (BBB) and coordinates cerebral blood flow in association with neuronal function. In cerebral gray matter, cellular constituents of the NVU include endothelial cells and associated pericytes, astrocytes, neurons, and microglia. Dysfunction of the NVU is a common feature of diseases that affect the CNS, such as ischemic stroke. High-level evaluation of these NVU changes requires the use of imaging modalities that can enable the visualization of various cell types under disease conditions. In this study, we applied our confocal microscopy strategy using commercially available labeling reagents to, for the first time, simultaneously investigate associations between endothelial cells, the vascular basal lamina, pericytes, microglia, astrocytes and/or astrocyte end-feet, and neurites in both healthy and ischemic brain tissue. This allowed us to demonstrate ischemia-induced astrocyte activation, neurite loss, and microglial migration toward blood vessels in a single confocal image. Furthermore, our labeling cocktail enabled a precise quantification of changes in neurites and astrocyte reactivity, thereby showing the relationship between different NVU cellular constituents in healthy and diseased brain tissue. The application of our imaging approach for the simultaneous visualization of multiple NVU cell types provides an enhanced understanding of NVU function and pathology, a state-of-the-art advancement that will facilitate the development of more effective treatment strategies for diseases of the CNS that exhibit neurovascular dysfunction, such as ischemic stroke.

## 1. Introduction

The neurovascular unit (NVU) is an anatomical and functional unit of cells associated with the cerebral vasculature that coordinates neuronal functions with vascular functions. In gray matter, the microvascular NVU consists of brain endothelial cells, pericytes, astrocytes, microglia, and neurons that interact to establish and maintain the blood–brain barrier (BBB) and tightly regulate cerebral blood flow through autoregulation and neurovascular coupling [1]. Furthermore, the perivascular space between cerebral blood vessels and the parenchyma is an area of rapid transport of extracellular fluid throughout the CNS [2,3,4]. The NVU must function properly to maintain central nervous system (CNS) homeostasis through its barrier and clearance properties, as well as rapidly increase blood flow to activated regions of the CNS via neurovascular coupling. Death or dysregulation of cellular components comprising the NVU is associated with many CNS disease states such as ischemic stroke. Indeed, restoring NVU functionality is a strategic goal for stroke treatment, as well as for other CNS diseases exhibiting NVU impairment [5,6,7].

At the NVU, cerebral endothelial cells (EC) form a thin monolayer of cells that line the inner surface of blood vessels. Brain ECs form the BBB and exhibit a phenotype characterized by a high transendothelial electrical resistance, low rates of transcytosis, and low paracellular permeability relative to most endothelial cells outside of the CNS. Tight junction protein complexes form physical seals between ECs, which greatly restrict the paracellular passage of polar molecules, ions, and macromolecules from the blood into the CNS [6,8,9,10]. A number of different transporters, metabolic enzymes, ion channels, and ion transporters are expressed at the BBB which further regulate CNS homeostasis by importing beneficial substances (e.g., glucose) into the CNS while restricting harmful xenobiotics from entering the CNS [10,11]. ECs also express various receptors capable of responding to neurotransmitters and signaling molecules released by other cell types of the NVU following physiological or pathological stimuli. Pericytes and astrocyte end-feet support the establishment and maintenance of endothelial barrier properties in the CNS.

Pericytes are a diverse population of mural cells that share a thin basement membrane with the abluminal surface of microvascular ECs and possess cytoplasmic processes, which tightly wrap around the endothelial cells of pre-capillary arterioles and capillaries [12]. In the CNS, pericytes play an important role in angiogenesis, blood-flow regulation, and the formation and maintenance of the BBB [13,14,15,16,17,18]. Pericytes and endothelial cells share membranous interdigitations called “peg and socket” interactions that allow for direct physical and biochemical communication between the two cell types, in addition to the paracrine signaling that occurs between these cells due to their close proximity [19]. Death or dysfunction of the pericytes can induce changes in BBB permeability and blood flow dysregulation and are increasingly recognized as an important factor in neurovascular pathologies [20].

Glial cell components of the NVU include astrocytes and microglia. Astrocytes are heterogeneous, star-shaped glial cells with a highly branched morphology that have been shown to secrete molecules and vesicles critical for tight junction formation in endothelial cells, BBB maintenance, and pericyte differentiation [10,21,22,23,24]. Another important role for astrocytes involves the regulation of water and ion exchange across the BBB [25]. Additionally, astrocytic Ca^2+^ fluctuations are involved in modulating neurovascular coupling [26,27]. Located abluminally and in close proximity to pericytes are astrocyte end-feet, which are cytoplasmic processes that wrap around cerebral microvessels. The astrocyte end-feet nearly completely cover the microvessels, and their position within the NVU allows them to rapidly communicate with neurons, pericytes, and endothelial cells. Astrocytes undergo morphological, molecular, and functional remodeling and become “reactive” in response to pathological stimuli [28]. Microglia are the most prominent immune cells of the CNS, with about one-third of these highly motile cells associated with blood vessels in a juxtavascular position [29,30]. Vessel-associated microglia are positioned in a location that allows them to communicate with neurons and vascular components of the NVU. Microglia have been shown to affect BBB integrity in response to CNS stress or injury [31,32,33]. Additionally, microglia are capable of modulating vascular structure, blood flow, and neurovascular coupling [30,34]. The activation of microglia in response to CNS infection or injury results in the release of inflammatory mediators and enhanced phagocytic activity and is implicated in the pathogenesis of many CNS disorders [35].

Neurons are electrically excitable cells that construct the fundamental functional units of the CNS. Restricted groups of neurons are able to produce hemodynamic responses along the entire cerebrovascular tree in order to increase blood flow to activated regions of the CNS. Neurons can initiate and modulate neurovascular coupling by releasing neurotransmitters that generate a vasoactive response from other cell types at the NVU when required [1]. Neural activity has been shown to regulate the structure of vascular networks in the brain [28]. Neurovascular dysfunction is frequently associated with CNS diseases exhibiting neurodegeneration [5].

A functional NVU requires tightly coordinated signaling between the different constituent cell types. The location of pericytes, astrocytic end feet, microglia, and neurites proximal to the vasculature is essential for signaling to occur rapidly and correctly. Changes in the spatial or morphological relationships between these components are frequently observed in neurological disease states and can alter functional aspects of the BBB and neurovascular coupling. In most microscopic analyses of the NVU, only one or two cell types are observed in a single image while the other cellular components are ignored or analyzed in separate images. The ability to simultaneously image additional components of the NVU is necessary to gain further understanding of the spatial, morphological, and functional relationships between different components of the NVU.

Here, we applied our high-resolution confocal microscopy approach to simultaneously image endothelial cells, pericytes, astrocytes, microglia, and neurites in a single fixed brain specimen. We have directly compared the “state” of the neurovascular unit in healthy rat brain tissue slices to rats that have been subjected to middle cerebral artery occlusion (MCAO) with subsequent reperfusion. Our analyses resulted in the observation of morphological and pathological changes that are associated with the NVU after an ischemic insult (i.e., glial activation, neurite loss). These data, obtained from different brain regions of healthy and/or diseased tissue, will help to advance the field of neurovascular biology by revealing intercellular relationships between different cell types of the NVU. These results will facilitate an improved understanding of changes to neurovascular anatomy in the context of neurological diseases such as ischemic stroke. Such information can be directly applied to the development of novel strategies to protect the NVU and optimize health for individuals afflicted with CNS pathologies.

## 2. Methods

### 2.1. Animals and Ethical Approval

Animal protocols were approved by the University of Arizona Institutional Animal Care and Use Committee (Protocol #11-252; Approval Date: 3 March 2020) and were conducted in compliance with both National Institutes of Health and Animal Research: Reporting In Vivo Experiments (ARRIVE) guidelines. Male Sprague Dawley rats (240–280 g) were purchased from Envigo (Madison, WI, USA). Animals were housed under controlled conditions (22.2–22.4 °C; 50% relative humidity; 12 h light/dark cycle) with free access to food and water for a minimum of seven days prior to tMCAO.

### 2.2. Transient Middle Cerebral Artery Occlusion (MCAO) Model

MCAO surgery was performed on six male rats according to our published method [36]. Briefly, surgeries were conducted using a Leica M80 surgical microscope (Leica Biosystems, Wetzlar, Germany). Rats were induced to a surgical plane of anesthesia using 4% isoflurane and maintained at 2.5–3% isoflurane anesthesia in O_2_ using a VetFlo™ vaporizer (Kent Scientific Corporation, Torrington, CT, USA). Continuous blood flow was measured using a laser-Doppler probe (Moor Instruments, Wilmington, DE, USA) that was placed on the skull directly above the region perfused by the left middle cerebral artery (MCA) (i.e., 2 mm posterior, 6 mm lateral to Bregma). Body temperature was maintained at 37 °C using a thermoregulated surgical pad (Scintica Instrumentation, London, ON, Canada) and continuously monitored using a rectal probe thermometer. Temperature maintenance is a critical consideration in MCAO studies as reduced body temperature can confound experimental results [37]. After shaving and aseptic preparation of the surgical site with betadine and 70% ethanol, bupivacaine (0.5% (*w*/*v*), 200 μL injection volume, s.c.; Millipore-Sigma, St. Louis, MO, USA) was administered for pain management. At this time, a ventral midline incision was made on the midline over the trachea. Careful blunt and sharp dissection was performed to expose the left common carotid artery (CCA), external carotid artery (ECA), and internal carotid artery (ICA) from the surrounding superficial fascia, digastric, sternohyoid, and sternomastoid muscles. After permanent ligation of the distal ECA and temporary ligation of the CCA, a small incision was made in the ECA and a silicone-coated intraluminal monofilament (0.33 mm tip diameter; Cat #503323PK10; Doccol Corporation, Sharon, MA, USA) was inserted and advanced approximately 2 cm into the ICA to occlude blood flow to the middle cerebral artery (MCA). Animals were excluded from analysis if they did not experience at least a 75% decrease in cerebral blood flow as measured via the laser-Doppler probe.

Following 90 min MCAO, the intraluminal filament was carefully removed to allow for the restoration of blood flow (i.e., reperfusion). A successful reperfusion was characterized by an increase of 70% or more of occluded blood flow. If this benchmark was not achieved, animals were excluded from further analysis. Sham control animals underwent the same surgical procedure as MCAO animals except for insertion of the intraluminal filament. Upon removal of the intraluminal filament, the incision site was closed with absorbable sutures and all animals received a local injection of bupivacaine (0.5% (*w*/*v*), 200 μL injection volume, s.c.) for post-operative pain management. All animals were placed in a socially housed recovery cage (≤4 animals per cage) with clean bedding, medicated wet food (Nutra-Gel Diet™ (F5769-KIT); formulated with 2.15 mg carprofen; Bio-serv, Flemington, NJ, USA) available *ad libitum* and thermal support via a heating pad.

### 2.3. TTC Staining

To validate the infarcted region in the immunofluorescence experiments, TTC (2,3,5-triphenyltetrazolium chloride) staining was used in accordance with a previously published method [36]. TTC staining provides a reliable colorimetric redox indicator of healthy versus damaged (i.e., both the infarction core and ischemic penumbra) brain tissue that results from an ischemic insult. Specifically, tissue containing viable mitochondria is stained dark red while infarcted brain tissue remains unstained (i.e., white). After 90 min MCAO followed by 22.5 h reperfusion, animals were anesthetized with ketamine/xylazine (K: 50 mg/kg; X: 10 mg/kg, i.p.) and decapitated using a guillotine. The brain was quickly harvested and sectioned using an ice-cold (4 °C) brain matrix, which enables the preparation of 1 mm brain slices in the rostral-to-caudal direction. The slices were then incubated in TTC (2% (*w*/*v*) in phosphate-buffered saline (PBS) at 37 °C) for approximately 5 min. Slices were then removed from the TTC stain, rinsed with PBS (pH 7.4), and imaged.

### 2.4. Laser Doppler Blood Flow

A MoorVMS laser Doppler system (Moor Instruments, Wilmington, DE, USA) was used for recording continuous blood flow readings during middle cerebral artery occlusion (MCAO). Sprague Dawley rats were induced to a surgical plane of anesthesia using 4% isoflurane and maintained at 2.5–3% isoflurane anesthesia in O2 using a VetFlo™ vaporizer (Kent Scientific Corporation, Torrington, CT, USA). A midline incision from the ears to parallel to the eyes was made and the skull exposed so that the coronal suture and sagittal suture are easily visible. The Doppler probe was placed on the skull directly above the left middle cerebral artery (MCA) region (MCA: 2 mm posterior, 6 mm lateral to Bregma) and secured using surgical grade adhesive (Moor Instruments, Wilmington, DE, USA).

### 2.5. Tissue Processing

Twenty-four hours following MCAO, rats were perfused with a glyoxal solution consisting of 3% glyoxal (Sigma Aldrich #128465, St. Louis, MO, USA), 20% ethanol, and 0.75% acetic acid, pH 4.5. Brains were removed and post-fixed overnight at 4 °C. The brains were then cryoprotected in 20% and 30% sucrose solutions in PBS and snap-frozen at −75 °C in isopentane on dry ice. Tissue sectioning was performed in a cryostat (Leica Biosystems) and 10 μm sections were mounted onto charged glass slides and stored at −80 °C until immunofluorescence analysis was performed.

### 2.6. Immunofluorescence Staining

Tissue sections were blocked for 15 min in True Black Supressor (Biotium, Fremont, CA, USA) followed by 15 min in Carbo Free Blocking Solution (Vector Laboratories, Newark, CA, USA) with 0.3% Tx-100. Sections were then incubated overnight at 4 °C in Carbo Free w/0.3% Tx-100 and rabbit anti-PDGFR-β (1:50; Thermo Fisher, Waltham, MA USA #MA5-15143), Alexa 488-Milli-Mark^®^ FluoroPan Neuronal Marker (1:100; Millipore Sigma #MAB2300X), Cy3-GFAP (1:100; Millipore Sigma, St. Louis, MO, USA #C9205), and DyLight 649-tomato lectin (1:1000; Vector Laboratories, Newark, CA, USA #DL-1178-1). Sections were then incubated in Carbo Free with 0.3% Tx-100 and Alexa 405 Plus goat anti-rabbit (1:200; Thermo Fisher # A48254) for 1 h at room temperature and coverslipped in ProLong Diamond (Thermo Fisher # P36970). When labeling Aquaporin-4 instead of GFAP, Alexa 488-Aquaporin-4 (Santa Cruz Biotechnology, Santa Cruz, CA, USA # sc-32739 AF488) and the Milli-Mark Pan Neuronal Marker antibody was labeled with CF555 Mix-n-stain labeling kit (Biotium) according to the manufacturer’s instructions. A list of labeling reagents used in this study can be found in Table 1.

### 2.7. Confocal Microscopy

Images were acquired using a Leica SP8 (Leica) confocal microscope equipped with HyD detectors and 405 nm, 488 nm, 552 nm, and 638 nm excitation lasers. Images were pseudo colored and 3D rendered using LAS X software (Leica). Some images were deconvolved using Leica HyVolution to achieve confocal super-resolution. Images were then processed and fluorescence was quantified using ImageJ software (NIH). The caudoputamen and motor cortex were chosen for analysis because these regions are perfused by the middle cerebral artery and were shown to be damaged by TTC staining.

### 2.8. Statistics

Data were analyzed using GraphPad Prism software (GraphPad Inc., La Jolla, CA, USA) and are presented as the mean ± s.d. *p* values were calculated using an un-paired Student’s *t*-test. A value of *p* < 0.05 was considered statistically significant.

## 3. Results

### 3.1. Fluorescence Staining and Confocal Imaging of the NVU

**Figure 1:**
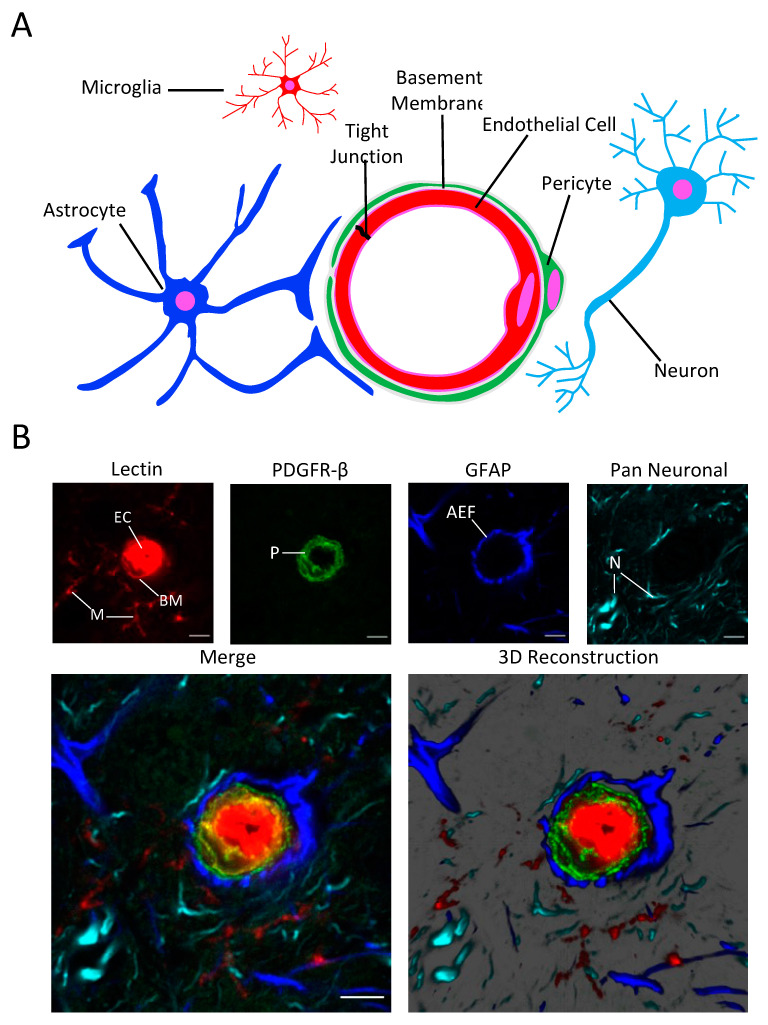
(**A**) Schematic showing cells and structures present at the NVU. (**B**) Confocal micrograph of a pre-capillary arteriole in the caudoputamen stained with the NVU labeling cocktail showing endothelial cells (EC), the basement membrane, (BM), microglia (M), pericytes (P), Astrocyte end-feet (AEF), and neutites (N). Scale bar = 5 μm.

**Figure 2:**
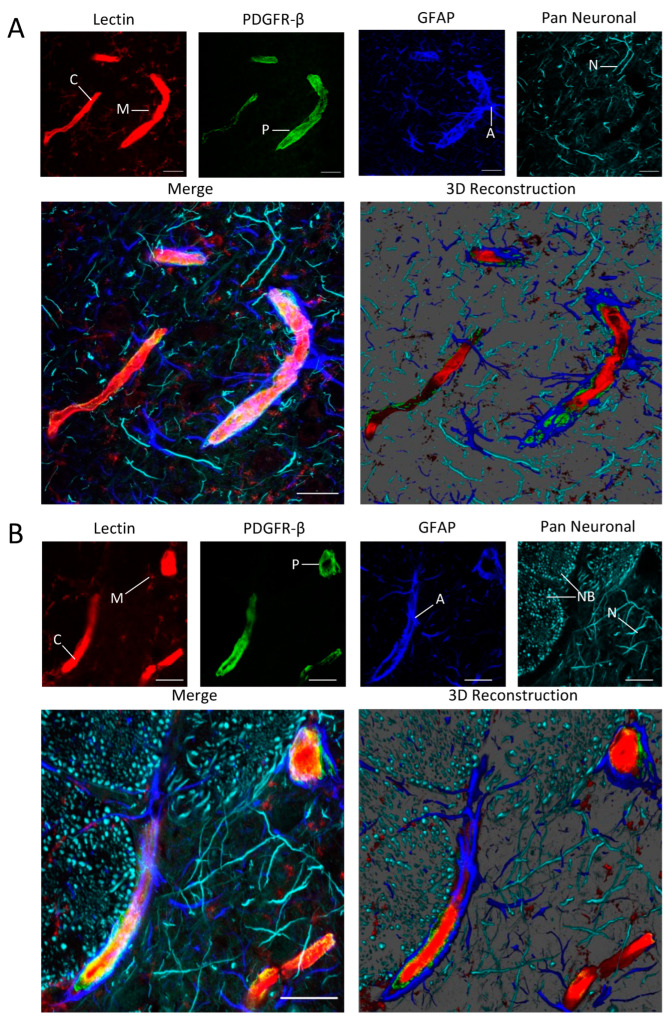
Images of regions in the cortex (**A**) and the caudoputamen (**B**) stained with the NVU labeling cocktail showing capillaries (C), microglia (M), pericytes (P), astrocytes (A), neurites (N), and nerve bundles (NB). Scale bar = 10 μm.

We imaged the NVU utilizing glyoxal fixation and a fluorescent labeling cocktail consisting of tomato lectin and antibodies to the pericyte marker PDGFR-β, the astrocyte marker GFAP, and the Milli-Mark Pan Neuronal marker. High resolution images were then acquired using laser scanning confocal microscopy. This allowed us to visualize several important structural components of the NVU at the level of the microvasculature. We were able to consistently observe the staining of the endothelial cells, the basement membrane, and microglia with tomato lectin, pericytes with anti-PDGFR-β, astrocytes with anti-GFAP, and neurites with the Pan Neuronal antibody in pre-capillary arterioles (Figure 1) and capillaries (Figure 2) within the same image.

**Figure 3:**
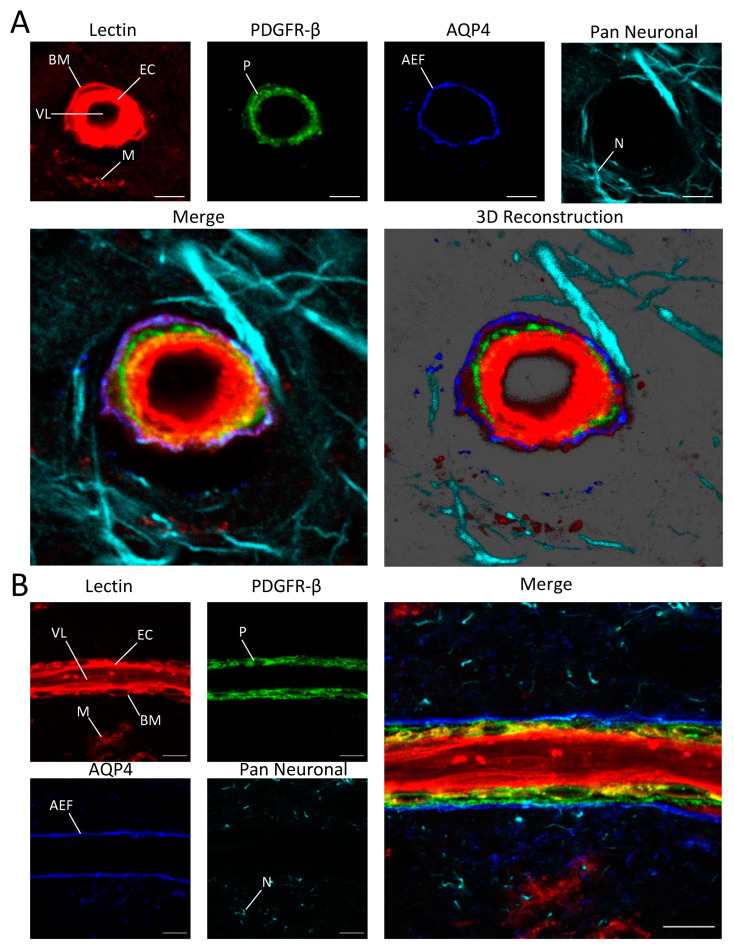
Images of capillary cross-section (**A**) and a longitudinal cross-section of a precapillary arteriole (**B**) in the cortex showing endothelial cells (EC), the casement membrane (BM), microglia (M), pericytes (P), astrocyte end feet (AEF), the vessel lumen (VL) and neurites (N). Scale bar = 5 μm in (B).

The astrocyte end-foot is the region of the astrocyte that is most important for signaling at the NVU because it wraps closely around blood vessels [38]. The water channel AQP4 is highly expressed at the astrocyte end-foot, and labeling AQP4 rather than GFAP is beneficial for studies focusing on this region of the astrocyte. For specific labeling of the astrocyte end-foot, we used an antibody that binds to AQP4 instead of GFAP and were able to clearly distinguish the endothelial cell layer from the pericyte layer and the astrocyte end-foot in microvascular cross-sections with our labeling cocktail (Figure 3).

### 3.2. Middle Cerebral Artery Occlusion (MCAO) Induces Changes in the NVU

**Figure 4:**
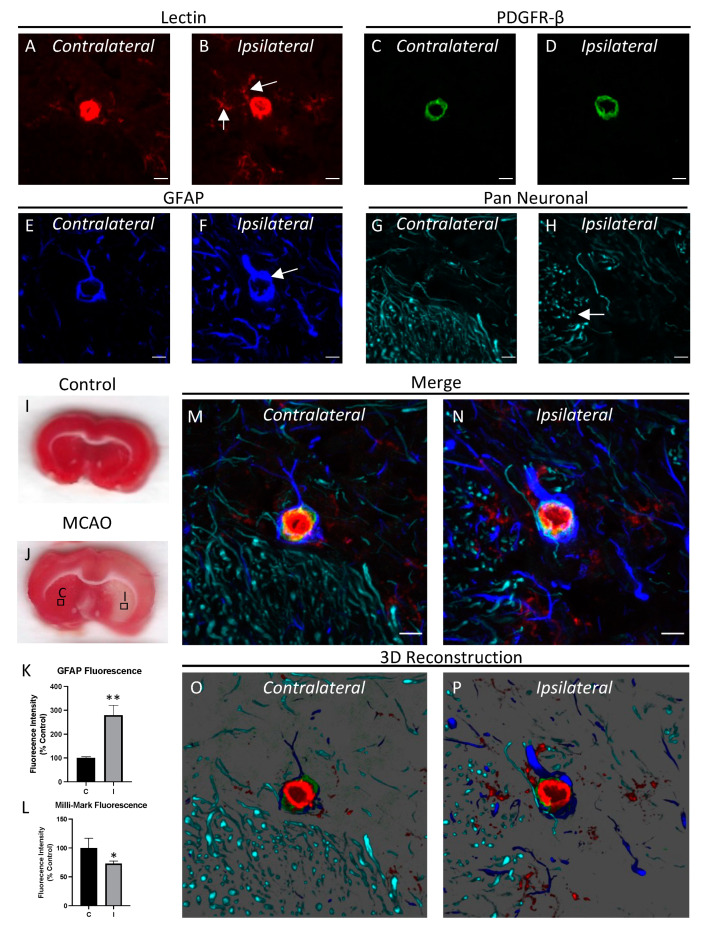
Ischemia-reperfusion injury induces changes in the NVU. The NVU was stained with lectin (**A**,**B**), anti-PDGFR-β (**C**,**D**), anti-GFAP (**E**,**F**) and the Pan Neuronal antibody (**G**,**H**), and capillaries from the caudoputamen in the hemisphere ipsilateral to where an MCAO was performed were compared to the contralateral hemisphere. The ipsilateral hemisphere shows an increased association of microglia with capillaries (arrows in **B**), an increase in GFAP fluorescence (arrow in **F**), and neurite loss (arrow in **H**) when compared to the contralateral side. Representative images of TTC-stained coronal sections from control (**I**) and MCAO (**J**) treated rats with boxes showing the approximate regions where the ipsilateral (**I**) and contralateral (**C**) images were acquired. Fluorescence intensity from the contralateral (**C**) and ipsilateral (**I**) regions was quantified for GFAP (**K**) and Milli-Mark (**L**) staining (*n* = 6). Merged images (**M**,**N**) and 3D reconstructions (**O**,**P**) are shown below. Images are representative of randomly chosen regions of interest in the caudoputament from 6 different rats. Scale bar = 5 μm; * *p* < 0.05; ** *p* < 0.01.

**Figure 5:**
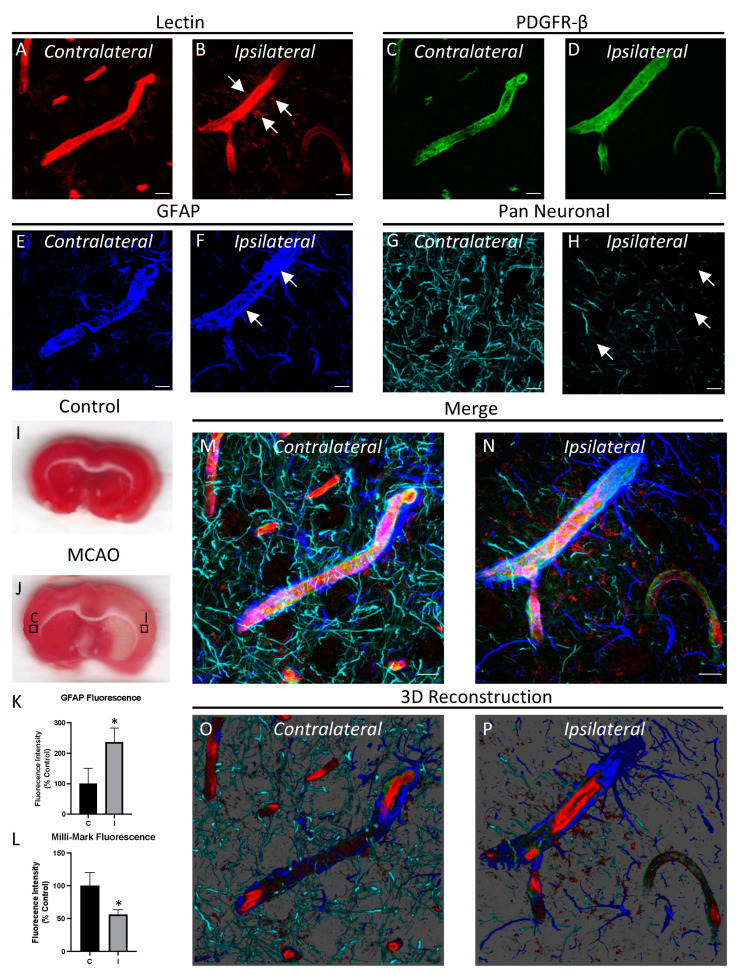
Ischemia-reperfusion injury induces changes in the NVU. The NVU was stained with lectin (**A**,**B**), anti-PDGFR-β (**C**,**D**), anti-GFAP (**E**,**F**) and the Pan Neuronal antibody (**G**,**H**), and cortical microvessels in the hemisphere ipsilateral to where an MCAO was performed were compared to the contralateral hemisphere. The ipsilateral hemisphere shows an increased association of microglia with capillaries (arrows in **B**), an increased in GFAP fluorescence (arrows in **F**), and neurite loss (arrows in **H**) when compared to the contralateral side. Representative images of TTC-stained coronal sections from control (**I**) and MCAO treated rats (**J**) with boxes showing the approximate regions where the ipsilateral (**I**) and contralateral (**C**) images were acquired. Fluorescence intensity from the contralateral (**C**) and ipsilateral (**I**) regions was quantified for GFAP (**K**) and Milli-Mark (**L**) staining (*n* = 6). Merged images (**M**,**N**) and 3D reconstructions (**O**,**P**) are shown below. Images are representative of randomly chosen regions of interest in the somatosensory cortex from 6 different rats. Scale bar = 10 μm; * *p* < 0.05.

It is well-established that certain diseases such as ischemic stroke involve pathological changes of the NVU. We imaged the NVU after inducing ischemia for 90 min followed by 22.5 h of reperfusion. Ischemia was confirmed by laser Doppler blood flow (Supplementary Figure S1). Cell death and the infarct area was identified by TTC staining in the caudoputamen (Figure 4I,J) and cortex (Figure 5I,J). By comparing the ipsilateral hemisphere where ischemia was induced with the contralateral (control) hemisphere, we were able to observe changes in the NVU that are associated with the pathogenesis of ischemic stroke. Specifically, we observed an increase in the perivascular microglia in the ipsilateral hemisphere as compared to the contralateral hemisphere (Figure 4A,B and Figure 5A,B), suggesting microglial migration towards injured blood vessels [29]. In addition to microglial activation, astrocytes are known to react to ischemia-reperfusion injury by proliferating to areas of vascular damage and forming a glial scar between areas of damaged and healthy tissue [39]. A characteristic of astrocyte activation is the up-regulation of GFAP, the major intermediate filament composing the astrocyte cytoskeleton [40]. We observed astrocyte reactivity after MCAO by showing an increase in GFAP fluorescence in the ipsilateral vs. the contralateral hemisphere (Figure 4E,F and Figure 5E,F). We also observed neurodegeneration following MCAO as evidenced by fewer neurites in the parenchyma of the ipsilateral side compared with the contralateral side (Figure 4G,H and Figure 5G,H). GFAP fluorescence was quantified and shown to be significantly increased in the ipsilateral vs. contralateral region for the caudoputamen (Figure 4K; n = 6; *p <* 0.01) and the cortex (Figure 5K; n = 6; *p* < 0.05). Milli-Mark (neurite) fluorescence was quantified and shown to be significantly decreased in the ipsilateral vs. contralateral region for the caudoputamen (Figure 4L; n = 6; *p <* 0.05) and the cortex (Figure 5L; n = 6; *p* < 0.05). Taken together, we simultaneously observed with high resolution multiple early NVU changes following ischemic stroke, including microglial migration towards blood vessels, astrocyte activation, and neurite loss.

## 4. Discussion

It is important that the NVU properly functions so cerebral blood flow can be precisely regulated, the BBB can be established and maintained, and extracellular fluid can rapidly and efficiently transfer solutes along perivascular spaces. Coordination between endothelial cells, pericytes, astrocytes, neurons, and microglia are necessary for efficient NVU function. Alterations in physiological signaling or cell death of NVU components are a feature of many diseases affecting the CNS. The visualization of NVU cellular components in health in disease has been reported throughout the peer-reviewed scientific literature; however, conventional imaging protocols typically label only two or three cell types. This approach greatly limits the quantity of information that can be obtained from a single image. Indeed, most antibodies used for immunofluorescence studies are generated in mice or rabbits, which limits the number of targets that can be imaged using secondary antibodies for indirect immunofluorescence. Our imaging approach constitutes a significant advancement in the field, as we have addressed this limitation by labeling endothelial cells and microglia with lectin in combination with labeled primary mouse antibodies that bind to astrocytes and neurite markers. This strategy allowed us to simultaneously image endothelial cells, pericytes, the endothelial basement membrane, astrocytes, microglia, and neurites, thereby optimizing the quantity of data that can be generated by a single fluorescent confocal image. Furthermore, our approach facilitated the evaluation of changes in multiple cell types associated with specific blood vessels. Therefore, we were able to generate a data set that provided critical information on NVU changes in the setting of ischemic stroke, results that can inform the discovery and/or development of novel treatment paradigms for this neurological disease.

We used tomato lectin in our labeling cocktail because of its ability to bind to both endothelial cells and microglia. Lectins are carbohydrate binding proteins and lectin from *Lycopersicon esculentum* (tomato lectin) has been shown to bind N-acetyl lactosamine and/or N-acetyl glucosamine oligosaccharides present on the glycocalyx, plasma membrane, and basal lamina of endothelial cells, in addition to the plasma membrane of microglia [41,42,43,44,45]. In our preliminary studies, we compared brain tissue perfusion-fixed with paraformaldehyde (PFA) or glyoxal with fresh-frozen sections fixed with methanol and found that tomato lectin yielded the best staining of endothelial cells and microglia on glyoxal-fixed tissue (Supplementary Figure S2). Glyoxal is an aldehyde that cross-links proteins by a mechanism similar to PFA; however, glyoxal-fixed brain tissue has been shown to yield superior immunofluorescence to paraformaldehyde-fixed brain tissue for a number of different antigens [46]. Glyoxal has also recently been shown to be superior to PFA when labeling endothelial cells, pericytes, and tight junction proteins. Therefore, our labeling procedure was performed on tissue fixed with glyoxal [47]. In addition to tomato lectin, we used antibodies to PDGFR-β for pericytes [48], GFAP for astrocytes [49] and aquaporin-4 for astrocyte end-feet [50]. We labeled neurites with the Pan Neuronal Milli-Mark antibody because it labeled more neurites than antibodies to microtubule associated protein 2 (MAP2) or Beta-tubulin III in our preliminary experiments.

A primary objective of our study was to image the NVU following MCAO with reperfusion in brain tissue isolated from male Sprague Dawley rats. Several different pathological hallmarks are associated with the NVU during ischemic stroke, including but not limited to glial activation, scar formation, neurodegeneration, and cell loss of endothelial cells and pericytes [20,29,51,52,53]. Using our labeling cocktail, we visualized several different early pathological changes in a single image following experimental stroke. We observed increased GFAP fluorescence indicating astrocyte activation [39], migration of microglia towards blood vessels [29], and neurodegeneration [51] as seen by neurite loss with the pan neuronal antibody. Additionally, microglia had fewer ramifications and larger cell bodies following MCAO, which suggests a transition towards an activated state.

Quantification of the pan neuronal marker and GFAP indicated an inverse relationship between neurite loss and astrocyte activation in the infarct region, suggesting that astrocyte activation may contribute to or be in response to neurodegeneration following MCAO.

The changes seen in the present work highlight the hallmark immediate neurodegeneration that occurs at the onset of ischemic injury due to rapid ATP depletion [54]. In the hours following stroke, the pro-inflammatory response peaks at 6 h where both microglia and astrocytes are activated, producing cytokines such as interleukin 1 beta (IL-1β) and tumor necrosis factor alpha (TNF-α) [55,56]. These responses aid in the rapid proliferation of astrocytes to the site of injury and eventual formation of the glial scar, as well as the recruitment of immune cells such as leukocytes to the infarcted area. Changes in endothelial cells and pericytes were not observed because these changes occur at later time points (i.e., >24 h after reperfusion) than the ones we studied. Although changes in pericyte density and localization were not observed in this study, pericytes do modulate the blood–brain barrier post-injury. These cells are believed to secrete proteases such as matrix metalloproteinease 9 (MMP-9) to disrupt tight junction protein complexes at the endothelia as well as the binding of astrocyte end feet, thus increasing blood–brain barrier permeability [57,58]. Such BBB breakdown provides an opening for leukocyte infiltration. At longer time points, the inflammatory response switches from pro- to anti-inflammatory to aid in the repair of the injured tissue. During this phase of post-stroke recovery, pericytes are responsible for secreting vasogenic factors that support vascular sprouting, such as platelet-derived growth factor receptor-beta (PDGFRβ), transforming growth factor-beta (TGF-β), vascular endothelial growth factor (VEGF), and angiopoietin 1,2 [58,59]. The stabilization of the microvasculature later (i.e., weeks post-stroke) promotes neurogenesis. It has been shown that glial scar formation can inhibit these repair processes by preventing immune cell infiltration into the scar [60]. It is expected that changes in pericyte reactivity, endothelial cell density and glial scar formation would be apparent in future imaging studies at > 24 h after reperfusion.

When fluorescently labeling NVU components, careful consideration must be taken to determine the cellular markers that are most appropriate for imaging. Of particular significance, development, aging, and disease can all affect the expression levels of different molecular targets. For example, PDGFR-β expression has been shown to increase after MCAO at time points later than the ones we studied [53]. We chose to use GFAP and PDGFR-β as markers for astrocytes and pericytes because these are the two most widely used markers for these respective cell types, but other markers for these cell types may be used instead of or addition to GFAP and PDGFR-β, depending on the study. Additionally, there are regional differences in the expression of GFAP and astrocyte morphology, so it is important to compare images of the NVU between the same regions of the CNS [61]. The focus of this study was to image the NVU in microvessels of cerebral gray matter. It is important to note that oligodendrocytes are present at the neurovascular unit in white matter [62]. In larger cerebral blood vessels, such as arterioles, perivascular macrophages and fibroblasts are present and pericytes are replaced by smooth muscle cells [12,63,64]. These factors must also be considered when imaging the NVU in white matter or larger blood vessels.

Our simultaneous imaging of multiple NVU components provided a more complete representation of how a disease affects the entire NVU. To this end, we reported microglial migration, astrocyte reactivity, and loss of neurites after MCAO. Observing pathological changes in multiple cell types of the NVU can direct therapeutic discovery and the development of novel treatments for ischemic stroke. Indeed, therapeutics may be effective in promoting cell viability in some, but not all, NVU cell types, and these differences may not be readily apparent when analyzing only two or three cell types. Most therapeutics used to treat ischemic stroke have focused on preventing neuronal death but, to date, few drugs have achieved success in advanced clinical trials. While there are several potential reasons for these negative outcomes, one possibility is that therapies targeting only neurons (and not any other cell types of the NVU) are ineffective at preventing neurological dysfunction caused by ischemia. It is important for all the cell types present at the NVU to function properly so neurons can receive the appropriate amount of energy and nutrients from the blood. A functional breakdown of endothelial cells, pericytes, astrocytes, or microglia all have the potential to cause downstream dysfunction or death of neurons. Studying the changes of the entire NVU is necessary to fully understand the pathogenesis of ischemic stroke and other diseases displaying neurovascular dysfunction. Future work will consist of looking at the neurovascular pathology at later time points following ischemic stroke, examining how other CNS diseases affect the pathology of the NVU, and investigating the effects of potential therapies on cells that comprise the NVU in CNS disease models. Overall, our studies provide an improved understanding of how ischemic stroke affects the NVU, data that will lead to optimized treatments for CNS diseases that exhibit modulation and/or protection of the NVU structure.

## Figures and Tables

**Table 1:** Labeling reagents used and their cellular targets.

Labeling Reagent (Biological Source)	Target
DyLight 649-tomato lectin (tomato)	Endothelial cells, basal lamina, microglia
Anti-platelet derived growth factor receptor-β (PDGFR-β) (rabbit)	Pericytes
Alexa 488-Aquaporin-4 antibody (mouse)	Astrocyte end-feet
Cy3-glial acidic fibrillary protein (GFAP) antibody (mouse)	Astrocytes
Alexa 488-Milli-Mark FluoroPan Neuronal Marker or CF555-Milli-Mark Pan Neuronal Marker (mouse)	Neurites

## Data Availability

If reasonably requested or needed, data and samples/models will be made available for sharing to qualified parties provided that such a request does not compromise intellectual property interests, interfere with publication, or betray confidentiality. Data that are shared will include standards and notations required to accurately interpret the data, following commonly accepted practices in the field. Data and samples/materials will be available for access and sharing as soon as reasonably possible and no longer than two years after acquisition of the data.

## Supplementary Materials

The following supporting information can be downloaded at: https://www.mdpi.com/article/10.3390/cells12040645/s1, Figure S1: Laser Doppler blood flow in the region of the middle cerebral artery (MCA); Figure S2: Fixative effects on lectin staining.
