# Supplementary material for: High Resolution Multiplex Confocal Imaging of the Neurovascular Unit in Health and Experimental Ischemic Stroke
Source: Cells. 2023 Feb 17;12(4):645. doi: 10.3390/cells12040645 (PMC9954836; doi:10.3390/cells12040645)
Supplement: Supplementary file 1 [file cells-12-00645-s001.zip › cells-1915582-supplementary.pdf]

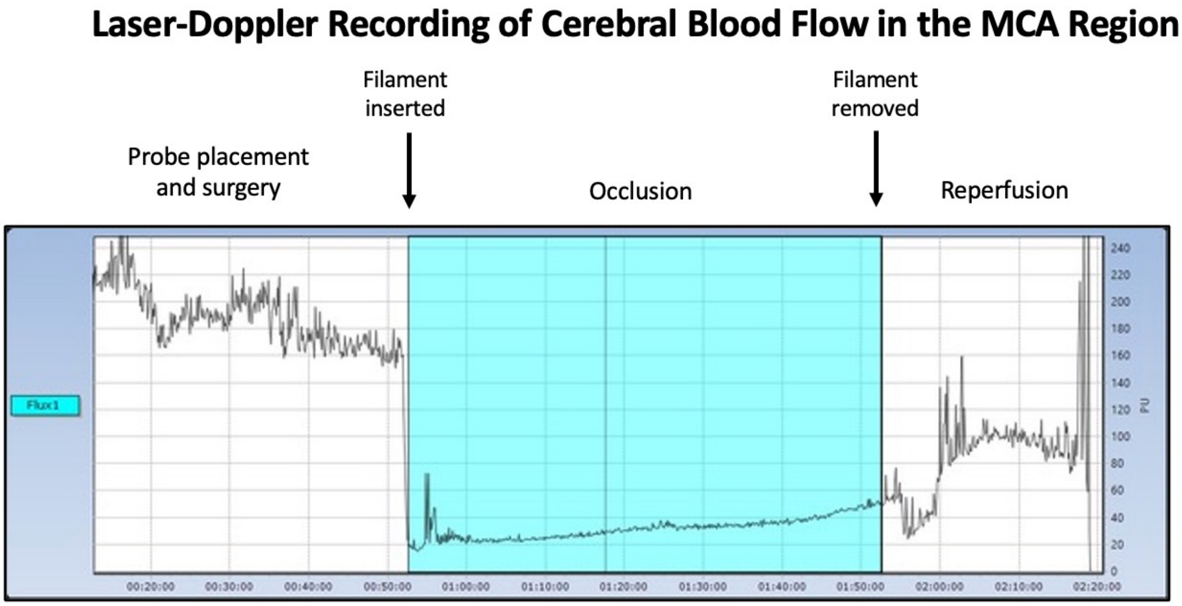

Supplementary Figure S**1.** Laser Doppler blood flow in the region of the middle cerebral artery (MCA). Highlighted in blue in the chart above, marks the occlusion period during the MCAO surgery. Approximately 80% reduction in blood flow was sustained during the occlusion period. Blood flow is restored during reperfusion, however, does not return to baseline levels due to ischemic injury.

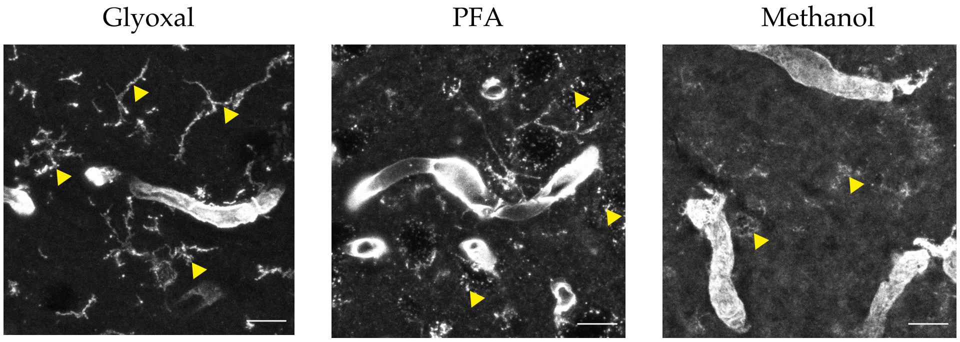

Supplementary Figure S2. Fixative effects on lectin staining. We examined the staining patterns of tomato lectin on tissue perfusion-fixed with glyoxal, 4% paraformaldehyde (PFA), and fresh-frozen tissue fixed with methanol. Glyoxal-fixation resulted in staining of microglia exhibiting ramified processes (triangles) and little background fluorescence. PFA fixation resulted in fewer identifiable microglia and non-specific, punctate background fluorescence (triangles). Methanol fixation resulted in weak microglial staining (triangles) with few ramifications.
